# Expert Consensus on Dry Needling Practices for Headache: An International Delphi Study Protocol

**DOI:** 10.3390/jcm14051740

**Published:** 2025-03-05

**Authors:** Thomas Perreault, Jan Dommerholt, César Fernandez-de-las-Peñas, Lars Arendt-Nielsen, Barbara Cagnie, Stefano Di Antonio, Matteo Castaldo

**Affiliations:** 1 Department of Physical Therapy, Wentworth Douglass Hospital, Dover, NH 03820, USA; 2 Myopain Seminars, Bethesda, MD 20814, USA; jan@myopainseminars.com; 3 Department of Physical Therapy, Occupational Therapy, Rehabilitation and Physical Medicine, Universidad Rey Juan Carlos, 28922 Madrid, Spain; cesar.fernandez@urjc.es; 4 Cátedra Institucional en Docencia, Clínica e Investigación en Fisioterapia-Terapia Manual, Punción Seca y Ejercicio Terapéutico, Universidad Rey Juan Carlos, 28922 Madrid, Spain; 5 Center for Neuroplasticity and Pain, SMI, School of Medicine, Aalborg University, DK-9220 Aalborg, Denmark; lan@hst.aau.dk (L.A.-N.); diantoniostefano@gmail.com (S.D.A.); matteo.castaldo@poliambulatoriofisiocenter.com (M.C.); 6 Department of Gastroenterology & Hepatology, Mech-Sense, Clinical Institute, Aalborg University Hospital, DK-9000 Aalborg, Denmark; 7 Steno Diabetes Center North Denmark, Clinical Institute, Aalborg University Hospital, DK-9000 Aalborg, Denmark; 8 Department of Rehabilitation Sciences and Physiotherapy, Faculty of Medicine, and Health Sciences, Ghent University, Campus Heymans, 9000 Ghent, Belgium; barbara.cagnie@ugent.be; 9 Department of Neuroscience, Rehabilitation, Ophthalmology, Genetics and Maternal Child Health, University of Genoa, 16132 Genoa, Italy; 10 Department of Medicine and Surgery, Clinical Psychology, Clinical Psychophysiology and Clinical Neuropsychology Labs., University of Parma, 43121 Parma, Italy

**Keywords:** dry needling, Delphi, headache, consensus

## Abstract

**Background**: Dry needling is increasingly utilized by clinicians in the treatment of patients with headaches. Although current evidence supports the use of dry needling for reducing headache pain, needling approaches are inconsistent among published studies, and no guidelines on dry needling for headaches have been established. **Methods**: A study will be conducted using the Delphi method, consisting of three rounds of questionnaires sent to an expert panel of clinicians and researchers. **Results**: To guide the development of the initial survey, we completed a literature review of articles related to dry needling for headaches. A steering committee will assess the initial survey items synthesized from the literature search and provide recommendations for the first and subsequent rounds of the study. Percentage agreement will be the primary measure throughout all rounds of this study. We define consensus to have been reached when 75% agreement is achieved. **Conclusions**: We seek to develop clinical recommendations that will guide research and treatment practices regarding dry needling for headaches. Having consensus-driven recommendations on dry needling for headaches will assist researchers in the design of future studies on this condition. In addition, having guidelines that clinicians can reference prior to the delivery of dry needling for headaches would benefit patient care.

## 1. Introduction

On any given day, it is estimated that 15.8% of the world population will experience a headache [1]. According to the International Headache Society, a multitude of headache phenotypes exists, classified as a primary or secondary headache disorder [2]. Primary headaches include migraine and tension-type headache (TTH), among others, with an estimated pooled prevalence in the global population of 14% (95% CI 12.9–15.2) and 26% (95% CI 22.7–29.5), respectively [1]. Even if its pathogenesis is incompletely understood, migraine is characterized by a cyclic sensitization of cortical and subcortical brain areas that reached its peak during the headache phase, when an increased activation of the trigeminovascular system has been observed [3]. Migraine is reported to be the most burdensome of all headaches and is ranked within the top ten leading causes of disability worldwide [4]. In fact, it is the leading cause of years lived with disability in people between the ages of 15 and 49 years old [5]. Migraine presents as headache attacks lasting 4–72 h of moderate or severe intensity and typically have a pulsating quality and unilateral location. They can be aggravated by routine physical activity and are accompanied by nausea, photophobia, or phonophobia [2]. On the other hand, TTH pain does not worsen with routine physical activity and does not feature nausea. Photophobia or phonophobia may be present in some chronic cases. In addition, TTH is typically bilateral and has a pressing or tightening quality with mild to moderate intensity, lasting minutes to days [2]. Cervicogenic headache (CGH) is a secondary headache disorder that occurs by pain referral of cervical origin to the head from upper cervical joints or muscles, among other structures [2,6,7]. Convergence of nociceptive afferents from the upper cervical region, with neurons also receiving input by afferents of the trigeminal nerve, may explain the phenomenon of referred pain to the head [6]. The prevalence of CGH is far less researched compared to migraine and TTH; however, previous studies estimate the prevalence of CGH to be as low as 0.17% in the general population [8] to as high as 4.1% [9]. An earlier study also reported that patients with CGH experience similar negative effects on health-related quality of life as do patients with migraine and TTH, most significantly in the domain of physical functioning [10]. Whilst headache types differ in diagnostic criteria, it is common for their clinical findings and symptomology to overlap in some sub groups of patients [11]. To further complicate the clinical headache diagnosis, one headache may co-exist with another, such as TTH and migraine [12].

Lifestyle factors such as poor sleep, low levels of physical activity, poor dietary habits, alcohol use and smoking, among others, may negatively impact headache characteristics [13,14]. Yet, educating individuals suffering from migraine, TTH or CGH to modify these lifestyle factors, when possible, and to keep an accurate headache diary may improve headache care [15]. In addition, headache types like migraine are associated with psychosocial comorbidities such as depression and anxiety, which add to the headache burden [16]. Consideration of these factors in the overall management of headache are being increasing investigated [12,13].

Cervical musculoskeletal impairments are prevalent in patients with migraine, CGH, and TTH [17,18,19]. These include increased pain sensitivity and dysfunction in the upper cervical spinal joints, restricted cervical range of motion, forward head posture, decreased function of the deep neck flexor muscles, reduced thoracic spine mobility, and trigger points in muscles of the head and neck [20,21,22,23,24]. A trigger point (TrP) is defined as a hypersensitive spot located within a taut band of skeletal muscle that is painful upon stimulation, and typically features referred pain and associated phenomena [25]. Tender points are also associated with primary and secondary headache types, commonly along the peri cranial and cervical region [26,27]. Like TrPs, tender points exhibit hyperalgesia and other signs reflective of central sensitization [28,29], but do not share the same pathophysiological features that are characteristic of TrPs, such as localized muscle contractures [30] and elevated concentrations of algesic and inflammatory substances [31]. Phenotypically, a TrP may exhibit nociceptive, nociplastic, neuropathic or mixed-type pain features in nature. That is, TrPs may be a primary source of pain or altered nociception, or secondary and comorbid with another disorder [25]. According to recent evidence, nociceptive input from TrPs in the craniofacial, craniomandibular and cervical region can also project to the trigeminocervical complex (TCC) and may precipitate headache pain via convergence of cervical and trigeminal afferents in the upper cervical spinal cord [32]. Trigger points are treatable musculoskeletal impairments, and therefore often targeted with various interventions in the clinical setting for the management of headache disorders [33].

Clinicians often use needling therapies for the treatment of TrPs in patients with headache. These include TrP injections using local anesthetics [33], botulinum toxin [34] or dry needling, where no pharmacological substance is used [35] and acupuncture [36]. Dry needling (DN) involves the insertion of solid monofilament needles through the skin and into various tissues of the body, most commonly into TrPs, to reduce pain and improve function [37]. Acupuncture is based on classical theories and principles of Traditional Chinese Medicine and involves the use of needles into acupuncture points to alter or regulate the flow of energy along meridian pathways [38]. Although DN and acupuncture differ in historical background and theories, both practices are commonly used for treating pain and share similar neurophysiological mechanisms [38]. Furthermore, many acupuncture points prescribed for treating headaches are in or around muscles of the head and neck that harbor TrPs [36,39].

De Pauw et al. reported consensual agreement across experts that impairments in muscle length/stiffness are good clinical indicators for the use of TrP therapy in patients with TTH in their Delphi study [40]. A previous study on patients with migraine reported that identification of TrPs in the head and neck muscles, specifically those TrPs eliciting migraine-like pain, has utility when planning to administer needling interventions [41]. Yet, DN may also target nerve innervation fields [42], periosteal structures and cervical entheses [43] for the management of headache pain. Dry needling is used by physicians, physical therapists, and chiropractors, among others [44,45], in many countries of the world for the treatment of neuromusculoskeletal disorders, including headache. Research on DN has grown over the past twenty years. In fact, a recent bibliometric analysis identified 458 articles published on the topic of DN for myofascial pain syndrome (MPS) alone, produced from 46 countries across the world [46]. Studies exploring the fundamental mechanisms of DN corroborate evidence for neurophysiological and biochemical effects that are elicited at peripheral [47,48], spinal [49,50], and supraspinal levels [37,51] that together serve to reduce nociception and modulate pain.

A recent umbrella review on DN concluded it is effective for reducing pain in the short term for musculoskeletal conditions associated with TrPs across all body regions, after analyzing nearly two hundred randomized controlled trials across thirty five systematic reviews [52]. In addition, DN was superior to the sham, placebo, and no intervention for reducing pain intensity. While no superiority was shown comparing DN to other interventions used in clinical practice, this review and others support using DN as an adjunct therapy [52,53]. For headaches, a previous review reported that DN reduced headache pain in all eight studies analyzed, including migraine [54]. Yet, Vázquez-Justes et al. reported high heterogeneity in the methodologies across DN studies, which rendered the authors unable to perform a meta-analysis. Furthermore, the authors supported the idea of developing specific protocols to increase consistency of methods across DN studies for headache [54]. Another recent meta-analysis reported that DN was not statistically better than other interventions for reducing headache pain intensity in the short term in patients with TTH (SMD −1.27, 95% CI = −3.56 to 1.03), CGH (SMD−0.41, 95% CI = −4.69 to 3.87), or mixed headaches (migraine and TTH) (SMD 0.03, 95% CI = −0.42 to 0.48). However, DN was shown to be effective for reducing disability and headache frequency compared to other interventions in patients with TTH and CGH [55]. According to Pourahmadi et al. most studies investigating dry needling for headache suffered from failure to report on allocation concealment and statistical heterogeneity. In addition, most studies poorly described DN procedures and failed to reference a specific DN approach. Moreover, no analysis regarding a specific DN technique on outcomes for any headache type could be performed due to the high variability in approaches used across studies [55]. It is conceivable that improved consistency of DN procedures across headache studies will allow researchers to observe if specific DN parameters impact study outcomes. For example, the physiological mechanisms of DN for pain modulation are influenced by needling dosage [48,50]. More specifically, the duration of needling is among the various dosage parameters and can influence the effect of DN on the autonomic nervous system [56]. Thus, if improved consistency in dosage parameters is achieved across studies using DN for headaches, researchers may begin investigating the impact of various DN dosages on outcomes for specific headache types.

In a recent DN review, the authors could not determine the impact of DN dosage on pain outcomes due to the wide variation in dosing variables and inconsistency in the needling techniques used between studies [57]. For example, only 2 of the 18 studies reported the number of needles used and only 4 of the 14 studies, which reported the elicitation of local twitch responses, specified how many occurred. As a result, the authors suggested reporting dosage parameters with specific recommendations on how to report DN technique, number of needles used per session, region treated, number of muscles and specific muscles treated per session, if a local twitch response (LTR) occurred (and if so how many), visit frequency and total visits [57]. Likewise, a survey study of physical therapists in the United States investigated DN practice patterns for the management of musculoskeletal pain and found significant variability in the use of needling techniques among respondents [58]. A more recent systematic review found considerable variation in and underreporting of DN procedures across more than twenty DN studies [59]. Consequently, the authors voiced concern over the potential impact this heterogeneity may have on the internal validity of many DN randomized controlled studies. Therefore, the Delphi process was recommended to assimilate a set of guidelines for DN [59]. Since studies investigating the use of DN for headaches are also widely inconsistent in needling approaches, and clinical guidelines on DN for headaches have not been established, a Delphi study would be an appropriate method to establish expert-based guidelines on DN for headaches [60].

In the healthcare setting, a Delphi study can generate consensus on a given research question through expert interviews using a questionnaire or other survey instrument. Typically, a structured questionnaire is issued over two to three rounds, undergoing several revisions after analyzing participant responses and feedback from the previous rounds. The aims of this Delphi study will be to develop a broad consensus on DN practices for the management of CGH, TTH, and migraine to inform practicing clinicians and further research studies on expert consensus-based practices for DN in these headaches. Additionally, it is our aim to contribute a study protocol that is consistent with established Delphi study guidelines to the clinical and research community involved in the use of dry needling for headache management.

## 2. Methods

### 2.1. Study Design

According to the Mass General Brigham (MGB) Institutional Review Board, the current project meets the criteria for exemption and was approved by the MGB Research Information Security Office (protocol #2024P001341). In addition, this protocol is registered on Open Science Framework (OSF) (https://doi.org/10.17605/OSF.IO/VBSY9). This study will implement a three-round Delphi design in accordance with the Guidance on Conducting and Reporting Delphi Studies (CREDES) (see Table S1) and other recommendations [61,62,63] along with direction from published Delphi studies in rehabilitation [18,40,64,65]. This study will consist of three rounds of online questionnaires sent to an expert panel of clinicians and researchers, both nationally and internationally. A systematic literature review of articles related to DN for headache was initially performed across several databases to inform the selection of survey items. A combination of closed and open-ended questions was developed to guide the first round of the survey. A steering committee has been established, and prior to the production phase of the Delphi, the committee will assess the study methodology, assess the initial survey items synthesized from the literature search, and provide any further recommendations for items to inform the first round, and subsequent rounds, of the Delphi study. This expert committee consists of six academic and clinical experts from the following countries: Spain, Denmark, Italy, Belgium, and the United States. Each committee member has over ten years of clinical and academic experience, has declared no conflicts of interest and has published on the topics of headache and/or dry needling extensively. After collection of participants’ responses and feedback from the first round and other rounds, the questionnaire will be refined and reissued to progress toward probable consensus.

Research Electronic Data Capture (REDCap) will be used to create, issue, and assist with analyzing electronic questionnaires from each round. Study data will also be collected and managed using REDCap electronic data capture tools hosted by Mass General Brigham Research Computing, Enterprise Research Infrastructure & Services (ERIS) group. Research Electronic Data Capture is a secure, HIPAA-compliant, web-based application designed to support data capture for research studies [66,67].

**Figure 1:**
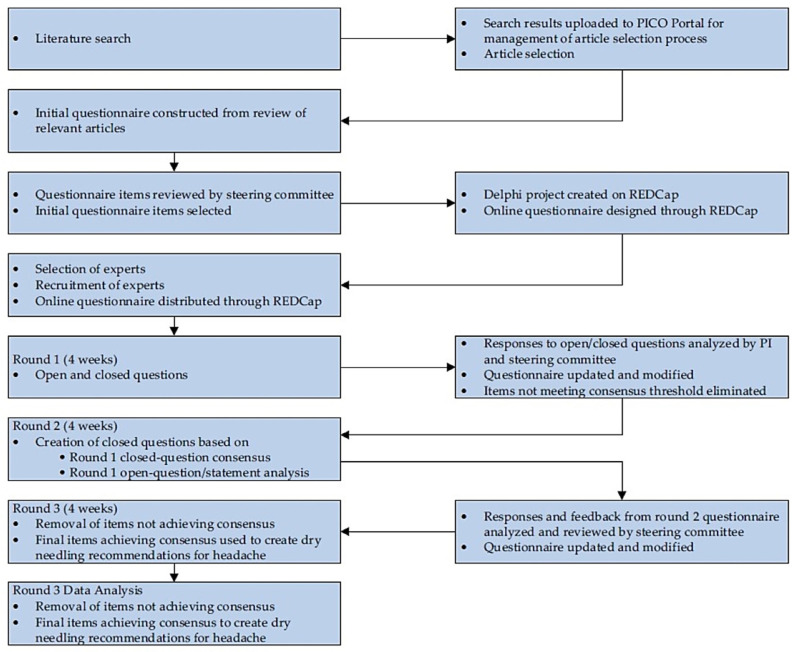
Delphi study schema.

Research data will be shared with the steering committee and a statistician for interpretation and analysis. In this study, data will be collected via electronic questionnaires delivered to the participants’ personal or institutional email address. Members of the steering committee, other than the PI, will have no direct contact with expert participants regarding this Delphi study process. Steering committee members will support the PI in the development of the initial questionnaire, help with data analysis and with formalizing future iterations of the questionnaire. All personal identifiers will be removed from the data sets and replaced with placeholder values. The controller of the data is the institution of the PI of the study. Finally, participants will be informed that the PI may reach out to them for further communications following the completion of the study when clarification of responses or feedback is needed for proper analysis of the study data, or for further data analyses related to this study only. Figure 1 presents the Delphi study schema.

### 2.2. Subject Selection

To capture a diverse sample, experts from a diverse range of institutions globally will be asked to participate. Eligible participants will include but not be limited to physicians, physical therapists, osteopaths, and chiropractors with at least 5 years of clinical experience in the management of headaches (CGH, TTH, and migraine) and at least 3 years of experience using DN for the treatment of patients with headache disorders. In addition, experts with 10% or more of their total treatment population that is headache related will be preferred [40]. Eligible individuals may also be academic researchers not currently in practice, but they must meet the defined clinical experience and DN experience requirements with headache management. Moreover, they must have achieved primary authorship or co-authorship of at least one relevant peer-reviewed publication on the topic of headache. The steering committee will be responsible for recruiting international experts, specifically experts identified through the literature search as authors, clinicians and/or academic professionals known to the members of the steering committee to have expertise in headache management, along with experts who are international DN instructors who meet the eligibility criteria.

There are no official guidelines available for researchers to use when selecting experts for a Delphi study, or determining appropriate sample size [68]. Based on a systematic review of the Delphi literature, Junger et al. confirmed that, at a minimum, the criteria used for the selection and recruitment of experts should be explained in the methodology, along with survey response rates and information related to the participants expertise [61]. For this study, we will lean on clinical and academic experience/knowledge related to headache and dry needling, along with authorship of peer-reviewed publications, which are commonly used criteria to define expertise in Delphi studies [68]. We aim to recruit and retain 30 experts from multiple countries, which is similar to the number of participants included in previous Delphi studies on the topic of headache [18,40] and DN [65]. Moreover, selecting an expert panel size larger than 30 may not improve the Delphi results and potentially contributes to lower survey response rates [69]. Importantly, a homogenous group of 30 experts with specialized knowledge and experience, in our case DN for headaches (CGH, TTH and migraine), is sufficient to obtain stable results from a Delphi survey [70]. Thirty participants that best represent the expert population most appropriate for this study will be chosen, following agreement to participate.

### 2.3. Subject Enrollment

A purposive sample of individuals will be invited via email to participate in this Delphi study. An email invitation will be sent to the potential panelists and will include a Delphi study fact sheet to provide information about the study. Following agreement to participate, a participant demographic questionnaire will be issued to assess eligibility for the study. Prior to the round one questionnaire, participants will answer questions related to basic demographics (age, sex, country of residence, current institution, or clinic) along with professional credentials, experience related to clinical practice, relevant academic experience, and DN experience for headache management. Upon confirmation of interest and eligibility, a REDCap survey will be sent electronically to the participant for rounds one through three. The principal investigator will be responsible for executing and managing all aspects of the survey process through REDCap. For participants who are non-English speaking, a translated version of the invitation email, and surveys for the study will be provided. Following consent to participate, each panelist will be asked to commit to participation in each round of the Delphi. Experts will be informed that participation is completely voluntary and there are no consequences for withdrawing from the study at any time or refusing to participate. Participants will be provided with a Delphi study information fact sheet explaining the type of data being collected and the reason for carrying out such a study. Potential participants will be provided with a clear explanation of the anticipated process, and an explanation that participation would last a period of 3–4 months.

### 2.4. Study Procedures

The initial survey items chosen for round one was supported by a systematic review of the literature on DN for migraine, TTH, and CGH. PubMed, CINAHL, and Web of Science databases were searched for articles published up to 17 October 2023. The criteria for inclusion were (1) articles written in the English language, (2) articles must either be a systematic review, meta-analysis, or randomized controlled trial investigating the effects of DN on migraine, TTH, or CGH; (3) articles that include outcome measures relating to either headache frequency, headache pain intensity, or disability. No limits were placed on the date of publications. Two reviewers who are Doctors of Physical Therapy, TP and JD were involved in the creation of the search syntax for each database, with the help of a Health Science Librarian who was also consulted in the literature search process. To optimize the chances of identifying relevant studies, we used a dual-reviewer title and abstract screening process [71,72]. The same two reviewers, TP, and JD, uploaded all search results data to the PICO Portal for automatic removal of duplicates and to carry out the process of screening titles and abstracts. Screening of titles and abstract together has been shown to have high precision in yielding relevant articles for systematic reviews [73]. In total, 9534 articles were identified, as shown in Table 1.

**Figure 2:**
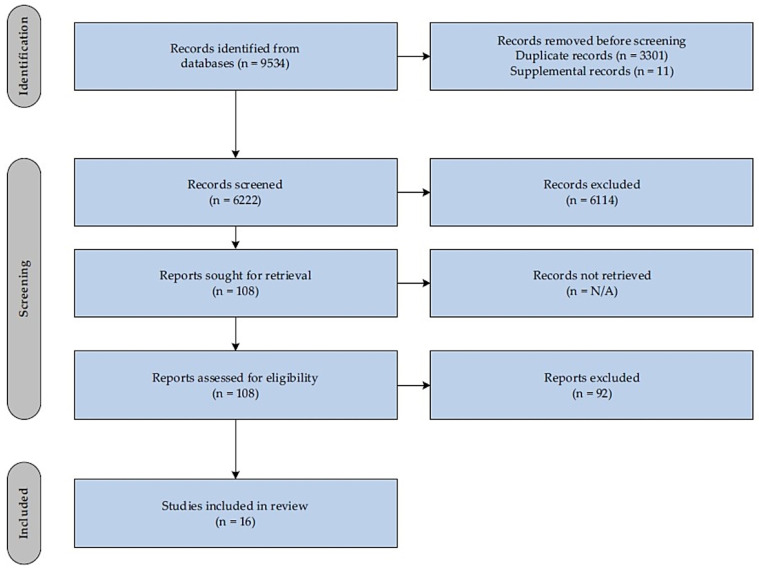
PRISMA flow diagram.

Following removal of duplicates, 6233 articles remained. After the screening of titles and abstracts, 6125 were excluded, leaving 108 for full-text review. Articles were excluded for the following reasons based on our inclusion criteria: wrong study design, not a dry needling study and article not in English. Finally, 16 articles fully met the inclusion criteria [7,42,43,54,55,74,75,76,77,78,79,80,81,82,83,84]. Figure 2 below presents the study selection process.

#### 2.4.1. Round One

Following the literature review, each of the sixteen studies was summarized by two reviewers, TP and JD, and the relevant methods and DN details of each study were organized thematically. Recording the details of the needling procedures across studies resulted in an archetypical set of dosage parameters on DN for headache that were used to develop the initial questionnaire. In addition, the overall treatment regimens, and the rationale behind the use of DN for headache were recorded. A previous DN Delphi study on plantar heel pain used the Standards for Reporting Interventions in Controlled Trials of Acupuncture (STRICTA) guidelines to develop their survey items [65], yet not all items recommended by the STRICTA guidelines are relatable to DN. Therefore, we followed recommendations from recent reviews on DN [57,59] in addition to STRICTA guidelines [85] when summarizing the studies, and to guide the development of the initial questionnaire, as shown in Table 2.

Two physical therapists, TP, and JD, developed the initial questionnaire. Topics identified from the study summaries in Table 2 were examined and translated into the initial survey items (see Table S2). Particular attention was given to the rationale and details of the needling procedures across studies to observe consistencies on how DN is administered in the treatment of headache. The initial survey items best reflected the common approaches on DN for headache. Each of the items included in the initial questionnaire related to DN rationale and DN dosage will be asked for each headache type (migraine, TTH and CGH). In addition, the initial survey will ask experts open-ended questions about any further DN considerations according to each headache type. Before distributing the initial survey to participants, the questionnaire will be pilot tested by three clinical and research experts from the steering committee.

Experts will rate their level of agreement for the included items using a 5-point Likert-scale, except for the initial open questions that will be included as part of the first round. Answers to open questions will be analyzed by the Principal Investigator (PI) and steering committee and converted to closed questions for the next survey round. After each item and open-ended question, there will be space for expert comments. Participant responses from the open-ended questions will be coded alongside each other and analyzed to identify concepts, categories and themes [88]. The PI will organize the data into a Microsoft Excel table and present it to the steering committee. An iterative process will ensue to determine what additional closed question items can be created for round two of the questionnaire based on the thematic analysis. Following the final round of the study, data from REDCap will be exported for statistical analysis.

We define that consensus has been reached when 75% percent of participants “strongly agree” or “agree” with an item. A percentage agreement value of 75% is commonly chosen for determining consensus among Delphi studies, according to a recent review [89]. Participants will not need to vote further on items achieving consensus in round one, and therefore items achieving consensus will not be included in the round two questionnaire. Items achieving 75% agreement or more for “disagree” or “strongly disagree” will be removed. Items that received an overall rating of “unsure” or that were rated as “strongly agree” or agree” by less than or equal to 50% of participants during the first and second round will require further investigation by the steering committee and items/questions will be amended for the next round. That is, if items that are believed to be important fall just below the threshold for consensus, the PI and steering committee will consider including these items as posteriori considerations provided that sufficient justification is provided. A table will be created to provide explanations for amended items moving into the next round. Following each round, the steering committee will review all questionnaire data, participant responses and comments, and the PI will make edits accordingly to prepare for the next survey round.

#### 2.4.2. Round Two

Themes originating from the open-ended responses and analysis of data from the structured questions of round one will guide the formation of the round two questionnaire. In round two, participants can modify their answers from round one based on results and feedback from that round. Participants will be able to see the amount of consensus on statements from the previous round, and where they answered compared to the group. Participants will not need to vote further on items achieving consensus in this round, and therefore items achieving consensus will not be included in the round three questionnaire. Items achieving 75% agreement or more for “disagree” or “strongly disagree” will be removed. Items that received an overall rating of “unsure” or that were rated as “strongly agree” or “agree” by less than or equal to 50% of participants during the first and second round will require further investigation by steering committee and items/questions will be amended for the next round. A table will be created to provide explanations for amended items moving into round three.

#### 2.4.3. Round Three

In round three, participants can modify their answers from round two based on results and feedback from that round. Participants will be able to see the amount of consensus on statements from the previous round, and where they answered compared to the group. If consensus is still not reached on a particular item, items will be reviewed and revised according to the criteria by the PI and steering committee and a fourth round will be performed. If all items have reached consensus according to our methods after round three, the questionnaire process will be complete. However, a fourth round may be performed to investigate expert agreement on an official list of recommendations for DN in headache management comprising all consensual items.

### 2.5. Statistical Analysis

Standard descriptive statistics will be used to describe the demographic characteristics of participants based on the first group of questions of the round one questionnaire. Anonymous answers from the open-ended questions following round one will be analyzed thematically as described in the study procedures section of this protocol. Percentage agreement calculations will be the primary measure throughout all rounds of this Delphi study [90]. However, depending on the data, measures of dispersion and central tendency may be used in place of or in addition to percentage agreement, using Interquartile Range (IQR) and/or Median, respectively, as these measures are also widely used in Delphi studies to define consensus [68]. Data will be analyzed using SPSS 30.0.0 or other statistical software at the end of each round. Wilcoxon rank-sum tests will be used for the final round to evaluate stability and consistency of responses between the final two rounds, with the aim of moving toward consensus [68]. The response rates of participants will also be measured following each round, which is increasingly investigated in Delphi studies [60,62].

## 3. Results

We plan to present our results through high-impact publication for the benefit of healthcare professionals, researchers, and patients. Outcomes will be displayed quantitatively (i.e., percentage agreement calculations, and possibly IQR and Median) and qualitatively. At the time of submission of this Delphi study protocol, the first round of this study is planned to begin on or before 1 March 2025.

## 4. Discussion

Dry needling has steadily evolved in clinical practice since its appearance in the literature as far back as the 1940s. Research interest in DN has grown substantially over the last 30 years, with studies investigating the use of DN for headache emerging in the early to mid-1980s [78,91]. At present, a recent meta-analysis study now supports DN as an effective intervention for reducing headache frequency and intensity across a range of headache types, with follow-ups of at least up to 3 months [92].

This study protocol describes the research design for a three, potentially four, round Delphi study to reach expert consensus for recommendations on the use of DN in headache management. The Delphi technique is a firmly established methodology for pursuing consensus on topics related to healthcare, and formulating recommendations based on expert opinion [60,69]. Having consensus-driven recommendations on DN for headaches would assist researchers in designing the methods and protocols for future clinical trials incorporating DN for headaches, and for this reason, a Delphi study will be performed. In addition, clinicians would benefit from having expert recommendations to reference prior to and during the delivery of DN treatments for patients with headaches, as no guidelines currently exist. After reviewing the literature on DN for headache, our observations are much like recent reviews on DN. That is, inconsistencies in DN procedures (i.e., dosage parameters and DN techniques) and practice patterns are prevalent across studies [57,59], especially those studies investigating DN for headache [55].

We also identified wide variations in treatment rationales across studies, with some studies referring to the peripheral mechanisms of DN and others leaning on more spinal or central mechanisms of action. In addition, the clinical indications for DN in headache management also fluctuate across studies. Most of the studies we reviewed administered DN to TrPs for treatment of headache, regardless of headache type. For example, Gildir et al. selected only active TrPs (TrPs that caused the clinical pain compliant when stimulated, i.e., at least part of the headache was reproduced with TrP stimulation) to be needled in patents with TTH [81,93]. Rezaeian et al. performed DN to active TrPs in the sternocleidomastoid muscle (SCM) in patients with migraine [76]. In contrast, Kamali et al. administered DN to TrPs in the suboccipital, temporalis, upper trapezius, or SCM muscles in patients with TTH, without specifying if TrPs elicited headache symptoms upon examination, or if they were latent [82]. A recent study, published after our review of the literature, analyzed the effects of DN on reducing the number of active TrPs in the cranial or facial muscles in patients with TTH, and reported a significant reduction in the total number of active TrPs, along with reductions in headache intensity [94]. None of the studies we reviewed specifically addressed latent TrPs (TrPs that are clinically dormant, are only painful when stimulated, and have similar characteristics as active TrPs) with DN in patients with headaches. Like active TrPs, latent TrPs in the muscles of the head and neck may be a source of local motor dysfunction and peripheral nociception that contribute to muscle pain as well as trigeminocervical and central sensitization [95,96]. It is conceivable that DN may be indicated not only for treatment of active TrPs, but also of latent TrPs in patients with headaches, especially since a recent study found that the pain-relieving mechanisms of DN are intrinsically related to reducing nociceptive input from TrPs, helping to attenuate peripheral sensitization [97]. However, regarding headache management, a multimodal approach that includes DN is recommended considering that nociplastic and neuropathic phenotypes of TrPs may be present, which are related to comorbid factors [25]. Nevertheless, according to a recent systematic review, it remains uncertain whether the TrP should be targeted for DN to be effective in the treatment of headaches [54]. Gaining expert consensus regarding dosage parameters, needling techniques, treatment rationales and clinical indications for the use of DN in headache would be valuable to research and treatment practices. In addition, the insights gained from this Delphi study may also be applicable to the management of various sub-types of headaches.

This Delphi study protocol does have some limitations. First, the questionnaire in its present form does not include items related to psychosocial factors like depression or anxiety, or lifestyle factors, which are relevant in the management of migraine, TTH and CGH. It is possible, however, that certain open-ended questions may lead to the inclusion of such items in other iterations of the questionnaire. Second, survey and questionnaire instruments used in Delphi studies may be subject to certain biases and misinterpretations, which may impact the credibility of the research being performed [98,99]. Third, because most of the research on DN for headache is focused on migraine, TTH and CGH, according to our review of the literature, not all headache types will be investigated in this study. Lastly, while we will include a diverse sample of experts from across the globe, there will be no specific limit to the number of potential experts from one region or country.

## 5. Conclusions

This study seeks to gather expert opinions regarding DN practices for headaches and synthesize them into group consensus via implementing a three-round Delphi study. Having expert guidelines and recommendations for DN practices for the management of cervicogenic, TTH, and migraine headaches would serve to inform researchers in designing the methods and specific protocols for future studies incorporating DN for these headache types. In addition, clinicians would benefit from having expert guidelines and recommendations to reference prior to and during the delivery of DN treatments for patients with headaches.

## Figures and Tables

**Table 1:** Search formulas for research databases.

Database	Search Terms	Date Searched	Records Found
PubMed	((“dry needling”[Title/Abstract] AND “Headache”[Title/Abstract]) OR “Migraine”[Title/Abstract] OR “Tension-Type”[Title/Abstract] OR “Tension-Type”[Title/Abstract] OR “Cervicogenic”[Title/Abstract]) AND ((clinicaltrial [Filter] OR meta-analysis [Filter] OR randomizedcontrolledtrial [Filter] OR systematicreview [Filter]) AND (humans [Filter]) AND (English [Filter]))	14 October 2023	4631
CINAHL	S3 = (AB “Dry Needling” AND AB Headache OR AB Migraine OR AB Tension-Type OR AB Tension Type OR AB Cervicogenic) AND (S1 OR S2) S2 = AB “Dry Needling” AND AB Headache OR AB Migraine OR AB Tension-Type OR AB Tension Type OR AB Cervicogenic S1 = TI “Dry Needling” AND TI Headache OR TI Migraine OR TI Tension-Type OR TI Tension Type OR TI Cervicogenic Search options: Limiters—Abstract Available; English Language; Research Article; Human Publication Type: Clinical Trial; Meta Analysis; Systematic Review; Randomized Controlled Trial Expanders—Apply equivalent subjects Search modes—Boolean/Phrase	17 October 2023	1813
Web of Science	(((((((((AB = (“Dry Needling”)) OR TI = (“Dry Needling”)) AND AB = (Headache)) OR TI = (Headache)) OR AB = (Migraine)) OR TI = (Migraine)) OR AB = (“Tension Type”)) OR TI = (“Tension Type”)) OR AB = (Cervicogenic)) OR TI = (Cervicogenic) Search refined by: Database: Web of Science Core Collection Language: English MeSH Headings: Humans Search within topic: Systematic Review, Meta analysis or Randomized Controlled Trial	17 October 2023	3090

**Table 2:** Summary of studies from the literature review.

Study	Study Type	Headache Type	Needling Rationale	Needling Details
				(A) Number of needle insertions, (B) location, (C) depth, (D) response sought, (E) type of needle stimulation, (F) needle retention, (G) needle type and (H) number of treatments
Venancio et al. 2009 [74]	RCT	TTH Migraine Mixed	Treat TrP to reduce peripheral sensitization and disrupt nociceptive input to the trigeminal system.	A. Multiple, not specifically stated B. TrPs in orofacial or cervical muscles, 1–3 TrPs C. Into the muscle (TrP), not specified D. LTRs until exhausted or pain reduced E. Manual, repeated insertion and withdrawal F. No retention G. Hypodermic (injection needle) H. Single treatment
Rezaeian et al. 2020 [76]	RCT	Migraine	DN can positively influence the trigeminal system and improve migraine headache by deactivating TrPs.	A. 8–10 needle insertions into TrPs B. TrPs in SCM C. Into muscle (TrP) D. Not specified, assume LTR based on Hong 1994 E. Fast-in-and-fast-out by Hong 1994 F. No retention G. Acupuncture needle, 25 mm × 25 mm length H. 3 treatments over 1 week, spaced 48 h apart
Sedighi et al. 2017 [7]	RCT	CGH	DN may improve local muscle blood flow to reduce ischemia and hypoxia, and LTR may reduce algesic substances.	A. Not specified B. TrPs in suboccipital and upper trapezius muscles C. Muscle depth or subcutaneous depth D. Not specified E. Not specified, manual stimulation F. Retention was 15 min G. Needle type not specified H. Single treatment
Togha et al. 2020 [77]	RCT	CGH	DN may promote changes in biomechanical and biochemical properties of TrPs to reduce headache symptoms.	A. Not specified B. TrP in SCM C. Into muscle (TrP) D. LTR to exhaustion E. Manual stimulation only F. None G. Acupuncture needle, 0.25 × 40 mm H. Four treatments delivered over 8 days, with one day interval between treatments
Hesse et al. 1994 [78]	RCT	Migraine	Inactivation of TrPs may reduce nociception contributing to headache.	A. Not specified B. TrPs, 1–3 per session, trapezius, rhomboids, and semispinalis capitis C. Into muscle (TrP) D. Response sought was not specified E. Manual needle stimulation F. No needle retention G. Acupuncture needle H. 6–8 treatments given over a 17-week period, with 1–3 weeks between treatments
Venancio et al. 2008 [75]	RCT	TTH Migraine Mixed type	Needling of TrP may interrupt reflex pain transmission, breaking the vicious TrP cycle. Endogenous pain modulation and positive effects on CNS.	A. Needle insertions not specified B. 1–3 TrPs per session that reproduced headache symptoms C. Into muscle (TrP) D. LTR until no longer able to elicit, or until muscle tautness is not perceived E. Manual stimulation only F. None G. Hypodermic H. Single DN treatment
Karakurum et al. 2001 [79]	RCT	TTH	DN may engage peripheral and spinal segmental mechanisms leading to pain relief and muscle relaxation.	A. Number of insertions not specified B. Six predesignated TrPs needled, two in splenius capitis, two in splenius cervicis and two in mid trapezius C. Into muscle (TrP) D. Response sought was not described E. Manual stimulation F. Needle retention for 30 min at each location G. A 30-gauge, 1 inch needle H. Four treatments
Mousavi-Khatir et al. 2021 [80]	RCT	CGH	DN to TrPs may reduce peripheral nociceptive input and activate spinal and central inhibitory pathways leading to reduction in CGH pain.	A. Number of needle insertions not specified B. Active TrPs in suboccipital, SCM and upper trapezius C. Into muscle (TrP) D. LTR to extinction E. Manual needle stimulation F. Needles were manipulated between 60 and 90 s G. Acupuncture needle, 25 mm × 30 mm H. Four treatments
Porter et al. 2022 [42]	RCT	CGH	Superficial DN over the trigeminal region may elicit neurophysiological effects that alter sensory input to the upper cervical dorsal horn leading to reduction in headache symptoms.	A. One needle insertion per location B. Innervation field of trigeminal nerve included locations near the supraorbital nerve, infraorbital nerve, mental nerve, and the auriculotemporal nerve C. Depth was superficial (<10 mm) D. Response sought was not specified E. Needle rotation was used, four rounds of 3–4 needle rotations F. Needle retention was 5–7 min G. Needles were 15 mm × 0.18 mm, acupuncture needles H. Single treatment session of superficial DN
Gildir et al. 2019 [81]	RCT	TTH	Mechanistic underpinning/rationale was not explained.	A. Number of needle insertions not specified B. Active TrPs in masseter, temporalis, frontalis, splenius cervicis and capitis, and sub-occipital muscles C. Into muscle (TrP) D. LTR was elicited to extinction in TrP E. Manual needle stimulation F. Needle retention was 20 min G. Acupuncture needles of 0.25 mm × 40 mm, 0.25 mm × 25 mm length H. Six DN treatments, 3× per week for 2 weeks
Kamali et al. 2019 [82]	RCT	TTH	DN of TrPs can reduce ischemia and improve blood flow to TrPs, leading to removal of sensitizing substances that contribute to central sensitization.	A. Number of needle insertions not specified B. TrP in sub-occipital, upper trapezius, temporalis and SCM C. Into muscle (TrP) D. LTR assumed, not specified E. Needle stimulation assumed manual based on reference F. Needle retention was not specified G. Needle type was not specified H. Three treatments over 1 week period
Dunning et al. 2021 [43]	RCT	CGH	Electrical DN and DN increases endogenous opioid levels, enhances microcirculation, and reduces pro inflammatory cytokines, potentially alleviating headache.	A. Number of needle insertions was 8–12, up to 4 thoracic region points allowed (potential of 16) B. Intramuscular locations included cranio-cervical, craniofacial, suboccipital, thoracic, shoulder gridle and first dorsal interossei muscles. Periosteal locations included posterior occipital rim and mastoid. Perineural locations included greater, lesser and third occipital nerve, supraorbital nerve, supra and infra trochlear nerve and artery, zygomatic-temporal nerve, and great auricular nerve C. Depth of insertion was 10 mm–30 mm D. Response sought was “mild to moderate” with electrical stimulation and needle rotation was used to elicit aching, tingling, deep pressure, heaviness, or warmth E. Needle stimulation included needle rotation and electrical stimulation Electrical parameters included 2 Hz frequency, 250 microsecond pulse width, biphasic continuous waveform at “mild to moderate” intensity F. Needle retention was 20 min G. Acupuncture needle; 0.18 × 15 mm, 0.25 mm × 30 mm and 0.30 mm × 40 mm length needles H. Up to 8 treatments, over 4 weeks
Vázquez-Justes et al. 2022 [54]	Systematic Review of RCTs	Migraine, TTH, CGH and Mixed type.	Eight studies were included for review. Two studies on TTH. Three studies on mixed headaches (TTH and Migraine). Two studies on CGH. One study on migraine headache. DN may induced local changes in skeletal muscle and lead to pain inhibition through peripheral and central mechanisms.	All studies included in this article are already described above, except for Patra et al. [86]. Patra et al. (2018) included patients with CGH. DN performed on TrPs in the trapezius, suboccipital and paraspinal muscles. Needle technique was not described, and 15–40 mm length needles were used. Session frequency not specified. The trapezius muscle was the only treated muscle in all studies. Three studies needled the suboccipital muscles and all other muscles varied between studies. Treatment schedules varied greatly between studies. Only two studies specified durations of treatment.
Lonzar et al. 2022 [83]	Systematic Review	Review of RCTs investigating invasive physiotherapy interventions for migraine.	Nine articles met the inclusion criteria, only one study used dry needling.	Only the study by Rezaeian et al. [76] used DN (see above). All other studies included acupuncture or percutaneous electrical needle stimulation.
France et al. 2014 [84]	Systematic Review	TTH, CGH	Three relevant studies were identified. Migraine studies were excluded. Two studies were RCTs, and one was a single case report.	Two studies investigated TTH, and the case report studied subjects with CGH. See Venancio et al. [75] and Karakurum et al. [79] above for needling details. Details of included case report were not extracted in this review.
Pourahmadi et al. 2021 [55]	Systematic Review and Meta-Analysis	Migraine, TTH, and CGH	Randomized trials and observations studies with control groups were included. Eleven studies were included. One study was in Korean, and two studies were not indexed on the database that we searched for the Delphi study. Four studies on CGH. Four studies on TTH. One study on migraine. Two studies on mixed population (migraine and TTH).	Dry needling method: 7 of the 11 studies did not reference the dry needling approach used. Local Twitch Response: Four studies reported that an LTR was elicited by dry needling of trigger points, and in the remainder it was unknown. Other: In 4 studies, the needles remained in TrPs for a period of 10–30 min Muscles treated Gildir et al. [81].: see above Hesse et al. [78]: see above Kamali et al. [82].: see above Karakurum et al. [79]: see above Patra et al. (2017, 2018) [86,87]: suboccipitals, paraspinals and trapezius Sedighi et al. [7]: see above Togha et al. [77]: see above Venancio et al. [74,75],: see above

Abbreviations: RCT = randomized controlled trial; LTR = local twitch response; TTH = tension-type headache; CGH = cervicogenic headache; SCM = sternocleidomastoid; TrP = trigger point; DN = dry needling; CNS = central nervous system.

## Data Availability

The original contributions presented in the study are included in the article/Supplementary Materials; further inquiries can be directed to the corresponding author.

## Supplementary Materials

The following supporting information can be downloaded at https://www.mdpi.com/article/10.3390/jcm14051740/s1: Table S1: CREDES checklist; Table S2: Initial survey items.

## References

[1] Stovner L.J., Hagen K., Linde M., Steiner T.J. (2022). The global prevalence of headache: An update, with analysis of the influences of methodological factors on prevalence estimates. J. Headache Pain.

[2] Headache Classification Committee of the International Headache Society (IHS) (2018). The International Classification of Headache Disorders, 3rd edition. Cephalalgia.

[3] Ashina M. (2020). Migraine. N. Engl. J. Med..

[4] Stovner L.J., Nichols E., Steiner T.J., Abd-Allah F., Abdelalim A., Al-Raddadi R.M., Ansha M.G., Barac A., Bensenor I.M., Doan L.P. (2018). Global, regional, and national burden of migraine and tension-type headache, 1990–2016: A systematic analysis for the Global Burden of Disease Study 2016. Lancet Neurol..

[5] Steiner T.J., Stovner L.J., Vos T., Jensen R., Katsarava Z. (2018). Migraine is first cause of disability in under 50s: Will health politicians now take notice?. J. Headache Pain.

[6] Bogduk N., Govind J. (2009). Cervicogenic headache: An assessment of the evidence on clinical diagnosis, invasive tests, and treatment. Lancet Neurol..

[7] Sedighi A., Nakhostin Ansari N., Naghdi S. (2017). Comparison of acute effects of superficial and deep dry needling into trigger points of suboccipital and upper trapezius muscles in patients with cervicogenic headache. J. Bodyw. Mov. Ther..

[8] Knackstedt H., Bansevicius D., Aaseth K., Grande R.B., Lundqvist C., Russell M.B. (2010). Cervicogenic headache in the general population: The Akershus study of chronic headache. Cephalalgia.

[9] Sjaastad O., Bakketeig L.S. (2008). Prevalence of cervicogenic headache: Vågå study of headache epidemiology. Acta Neurol. Scand..

[10] van Suijlekom H.A., Lamé I., Stomp-van den Berg S.G., Kessels A.G., Weber W.E. (2003). Quality of life of patients with cervicogenic headache: A comparison with control subjects and patients with migraine or tension-type headache. Headache.

[11] Zhang P. (2023). Which headache disorders can be diagnosed concurrently? An analysis of ICHD3 criteria using prime encoding system. Front. Neurol..

[12] Ashina S., Buse D.C., Bjorner J.B., Bendtsen L., Lyngberg A.C., Jensen R.H., Lipton R.B. (2021). Health-related quality of life in tension-type headache: A population-based study. Scand. J. Pain.

[13] Mingels S., Dankaerts W., van Etten L., Bruckers L., Granitzer M. (2021). Exploring multidimensional characteristics in cervicogenic headache: Relations between pain processing, lifestyle, and psychosocial factors. Brain Behav..

[14] Winter A.C., Hoffmann W., Meisinger C., Evers S., Vennemann M., Pfaffenrath V., Fendrich K., Baumeister S.E., Kurth T., Berger K. (2011). Association between lifestyle factors and headache. J. Headache Pain.

[15] Agbetou M., Adoukonou T. (2022). Lifestyle Modifications for Migraine Management. Front. Neurol..

[16] Duan S., Ren Z., Xia H., Wang Z., Zheng T., Li G., Liu L., Liu Z. (2023). Associations between anxiety, depression with migraine, and migraine-related burdens. Front. Neurol..

[17] Luedtke K., Starke W., May A. (2018). Musculoskeletal dysfunction in migraine patients. Cephalalgia.

[18] Luedtke K., Boissonnault W., Caspersen N., Castien R., Chaibi A., Falla D., Fernández-de-Las-Peñas C., Hall T., Hirsvang J.R., Horre T. (2016). International consensus on the most useful physical examination tests used by physiotherapists for patients with headache: A Delphi study. Man. Ther..

[19] del Blanco Muñiz J.Á., Sánchez Sierra A., Ladriñán Maestro A., Ucero Lozano R., Sosa-Reina M.D., Martín Vera D. (2024). Cervical impairments in subjects with migraine or tension type headache: An observational study. Front. Neurol..

[20] Anarte-Lazo E., Carvalho G.F., Schwarz A., Luedtke K., Falla D. (2021). Differentiating migraine, cervicogenic headache and asymptomatic individuals based on physical examination findings: A systematic review and meta-analysis. BMC Musculoskelet. Disord..

[21] Satpute K., Bedekar N., Hall T. (2023). Cervical neuro-musculoskeletal impairments in people with cervicogenic headache: A systematic review and meta-analysis. Phys. Ther. Rev..

[22] Liang Z., Galea O., Thomas L., Jull G., Treleaven J. (2019). Cervical musculoskeletal impairments in migraine and tension type headache: A systematic review and meta-analysis. Musculoskelet. Sci. Pract..

[23] Di Antonio S., Arendt-Nielsen L., Ponzano M., Bovis F., Torelli P., Finocchi C., Castaldo M. (2022). Cervical musculoskeletal impairments in the 4 phases of the migraine cycle in episodic migraine patients. Cephalalgia.

[24] Do T.P., Heldarskard G.F., Kolding L.T., Hvedstrup J., Schytz H.W. (2018). Myofascial trigger points in migraine and tension-type headache. J. Headache Pain.

[25] Fernández-de-Las-Peñas C., Nijs J., Cagnie B., Gerwin R.D., Plaza-Manzano G., Valera-Calero J.A., Arendt-Nielsen L. (2023). Myofascial Pain Syndrome: A Nociceptive Condition Comorbid with Neuropathic or Nociplastic Pain. Life.

[26] Aaseth K., Grande R.B., Lundqvist C., Russell M.B. (2014). Pericranial tenderness in chronic tension-type headache: The Akershus population-based study of chronic headache. J. Headache Pain.

[27] Castien R., Duineveld M., Maaskant J., De Hertogh W., Scholten-Peeters G. (2021). Pericranial Total Tenderness Score in Patients with Tension-type Headache and Migraine. A Systematic Review and Meta-analysis. Pain Physician.

[28] Ge H.Y., Wang Y., Danneskiold-Samsøe B., Graven-Nielsen T., Arendt-Nielsen L. (2010). The predetermined sites of examination for tender points in fibromyalgia syndrome are frequently associated with myofascial trigger points. J. Pain.

[29] Ashina M., Stallknecht B., Bendtsen L., Pedersen J.F., Schifter S., Galbo H., Olesen J. (2003). Tender points are not sites of ongoing inflammation -in vivo evidence in patients with chronic tension-type headache. Cephalalgia.

[30] Gerwin R.D., Cagnie B., Petrovic M., Van Dorpe J., Calders P., De Meulemeester K. (2020). Foci of Segmentally Contracted Sarcomeres in Trapezius Muscle Biopsy Specimens in Myalgic and Nonmyalgic Human Subjects: Preliminary Results. Pain Med..

[31] Shah J.P., Gilliams E.A. (2008). Uncovering the biochemical milieu of myofascial trigger points using in vivo microdialysis: An application of muscle pain concepts to myofascial pain syndrome. J. Bodyw. Mov. Ther..

[32] Goadsby P.J., Holland P.R., Martins-Oliveira M., Hoffmann J., Schankin C., Akerman S. (2017). Pathophysiology of Migraine: A Disorder of Sensory Processing. Physiol. Rev..

[33] Robbins M.S., Kuruvilla D., Blumenfeld A., Charleston L.t., Sorrell M., Robertson C.E., Grosberg B.M., Bender S.D., Napchan U., Ashkenazi A. (2014). Trigger point injections for headache disorders: Expert consensus methodology and narrative review. Headache.

[34] Wieckiewicz M., Grychowska N., Zietek M., Wieckiewicz G., Smardz J. (2017). Evidence to Use Botulinum Toxin Injections in Tension-Type Headache Management: A Systematic Review. Toxins.

[35] Dolina A., Baszczowski M., Wilkowicz W., Zieliński G., Szkutnik J., Gawda P. (2024). Trigger Point Therapy Techniques as an Effective Unconventional Method of Treating Tension Headaches: A Systematic Review. Healthcare.

[36] Liu L., Skinner M.A., McDonough S.M., Baxter G.D. (2016). Traditional Chinese Medicine acupuncture and myofascial trigger needling: The same stimulation points?. Complement. Ther. Med..

[37] Fernández-de-Las-Peñas C., Nijs J. (2019). Trigger point dry needling for the treatment of myofascial pain syndrome: Current perspectives within a pain neuroscience paradigm. J. Pain Res..

[38] Zhou K., Ma Y., Brogan M.S. (2015). Dry needling versus acupuncture: The ongoing debate. Acupunct. Med..

[39] Mata J., Sanchís P., Valentí P., Hernández B., Aguilar J.L. (2021). Treatment of headache disorders with acupuncture: A 6-year retrospective study. Acupunct. Med..

[40] De Pauw R., Dewitte V., de Hertogh W., Cnockaert E., Chys M., Cagnie B. (2021). Consensus among musculoskeletal experts for the management of patients with headache by physiotherapists? A delphi study. Musculoskelet. Sci. Pract..

[41] Calandre E.P., Hidalgo J., García-Leiva J.M., Rico-Villademoros F. (2006). Trigger point evaluation in migraine patients: An indication of peripheral sensitization linked to migraine predisposition?. Eur. J. Neurol..

[42] Porter M., Griswold D., Gargano F., Ickert E., Learman K. (2024). Immediate effects of superficial dry needling of the trigeminal nerve innervation field for participants with cervicogenic headache. A randomized controlled trial. Physiother. Theory Pract..

[43] Dunning J., Butts R., Zacharko N., Fandry K., Young I., Wheeler K., Day J., Fernández-de-Las-Peñas C. (2021). Spinal manipulation and perineural electrical dry needling in patients with cervicogenic headache: A multicenter randomized clinical trial. Spine J..

[44] Kalichman L., Vulfsons S. (2010). Dry needling in the management of musculoskeletal pain. J. Am. Board. Fam. Med..

[45] Ijaz N., Boon H. (2019). Evaluating the international standards gap for the use of acupuncture needles by physiotherapists and chiropractors: A policy analysis. PLoS ONE.

[46] Luo N., Li R., Fu B., Zeng Y., Fang J. (2023). Bibliometric and Visual Analysis in the Field of Dry Needling for Myofascial Pain Syndrome from 2000 to 2022. J. Pain Res..

[47] Liu Q.G., Liu L., Huang Q.M., Nguyen T.T., Ma Y.T., Zhao J.M. (2017). Decreased Spontaneous Electrical Activity and Acetylcholine at Myofascial Trigger Spots after Dry Needling Treatment: A Pilot Study. Evid. Based Complement. Altern. Med..

[48] Hsieh Y.L., Yang S.A., Yang C.C., Chou L.W. (2012). Dry needling at myofascial trigger spots of rabbit skeletal muscles modulates the biochemicals associated with pain, inflammation, and hypoxia. Evid. Based Complement. Altern. Med..

[49] Hsieh Y.L., Yang C.C., Liu S.Y., Chou L.W., Hong C.Z. (2014). Remote dose-dependent effects of dry needling at distant myofascial trigger spots of rabbit skeletal muscles on reduction of substance P levels of proximal muscle and spinal cords. Biomed Res. Int..

[50] Hsieh Y.L., Hong C.Z., Liu S.Y., Chou L.W., Yang C.C. (2016). Acupuncture at distant myofascial trigger spots enhances endogenous opioids in rabbits: A possible mechanism for managing myofascial pain. Acupunct. Med..

[51] Niddam D.M., Chan R.C., Lee S.H., Yeh T.C., Hsieh J.C. (2007). Central modulation of pain evoked from myofascial trigger point. Clin. J. Pain.

[52] Chys M., De Meulemeester K., De Greef I., Murillo C., Kindt W., Kouzouz Y., Lescroart B., Cagnie B. (2023). Clinical Effectiveness of Dry Needling in Patients with Musculoskeletal Pain-An Umbrella Review. J. Clin. Med..

[53] Sánchez-Infante J., Navarro-Santana M.J., Bravo-Sánchez A., Jiménez-Diaz F., Abián-Vicén J. (2021). Is Dry Needling Applied by Physical Therapists Effective for Pain in Musculoskeletal Conditions? A Systematic Review and Meta-Analysis. Phys. Ther..

[54] Vázquez-Justes D., Yarzábal-Rodríguez R., Doménech-García V., Herrero P., Bellosta-López P. (2022). Effectiveness of dry needling for headache: A systematic review. Neurol. (Engl. Ed.).

[55] Pourahmadi M., Dommerholt J., Fernández-de-Las-Peñas C., Koes B.W., Mohseni-Bandpei M.A., Mansournia M.A., Delavari S., Keshtkar A., Bahramian M. (2021). Dry Needling for the Treatment of Tension-Type, Cervicogenic, or Migraine Headaches: A Systematic Review and Meta-Analysis. Phys. Ther..

[56] Sillevis R., Van Duijn J., Shamus E., Hard M. (2021). Time effect for in-situ dry needling on the autonomic nervous system, a pilot study. Physiother. Theory Pract..

[57] Kearns G.A., Brismée J.M., Riley S.P., Wang-Price S., Denninger T., Vugrin M. (2023). Lack of standardization in dry needling dosage and adverse event documentation limits outcome and safety reports: A scoping review of randomized clinical trials. J. Man. Manip. Ther..

[58] Gattie E., Cleland J.A., Snodgrass S. (2020). A survey of American physical therapists’ current practice of dry needling: Practice patterns and adverse events. Musculoskelet. Sci. Pract..

[59] Myburgh C., Kildsgaard K., Damsgaard T., Corfixen K., Boyle E. (2021). Consistency of Dry-Needling Interventions Across High-Quality Randomized Trials: A Critical Systematic Exploration of Intervention Reporting and Fidelity. J. Manip. Physiol. Ther..

[60] Niederberger M., Spranger J. (2020). Delphi Technique in Health Sciences: A Map. Front. Public Health.

[61] Jünger S., Payne S.A., Brine J., Radbruch L., Brearley S.G. (2017). Guidance on Conducting and REporting DElphi Studies (CREDES) in palliative care: Recommendations based on a methodological systematic review. Palliat. Med..

[62] Spranger J., Homberg A., Sonnberger M., Niederberger M. (2022). Reporting guidelines for Delphi techniques in health sciences: A methodological review. Z. Evid. Fortbild. Qual. Gesundhwes.

[63] Hasson F., Keeney S., McKenna H. (2000). Research guidelines for the Delphi survey technique. J. Adv. Nurs..

[64] Luedtke K., Basener A., Bedei S., Castien R., Chaibi A., Falla D., Fernández-de-Las-Peñas C., Gustafsson M., Hall T., Jull G. (2020). Outcome measures for assessing the effectiveness of non-pharmacological interventions in frequent episodic or chronic migraine: A Delphi study. BMJ Open.

[65] Cotchett M.P., Landorf K.B., Munteanu S.E., Raspovic A.M. (2011). Consensus for dry needling for plantar heel pain (plantar fasciitis): A modified Delphi study. Acupunct. Med..

[66] Harris P.A., Taylor R., Thielke R., Payne J., Gonzalez N., Conde J.G. (2009). Research electronic data capture (REDCap)--a metadata-driven methodology and workflow process for providing translational research informatics support. J. Biomed. Inform..

[67] Harris P.A., Taylor R., Minor B.L., Elliott V., Fernandez M., O’Neal L., McLeod L., Delacqua G., Delacqua F., Kirby J. (2019). The REDCap consortium: Building an international community of software platform partners. J. Biomed. Inform..

[68] Shang Z. (2023). Use of Delphi in health sciences research: A narrative review. Medicine.

[69] de Villiers M.R., de Villiers P.J., Kent A.P. (2005). The Delphi technique in health sciences education research. Med. Teach..

[70] Akins R.B., Tolson H., Cole B.R. (2005). Stability of response characteristics of a Delphi panel: Application of bootstrap data expansion. BMC Med. Res. Methodol..

[71] Gartlehner G., Affengruber L., Titscher V., Noel-Storr A., Dooley G., Ballarini N., König F. (2020). Single-reviewer abstract screening missed 13 percent of relevant studies: A crowd-based, randomized controlled trial. J. Clin. Epidemiol..

[72] Waffenschmidt S., Knelangen M., Sieben W., Bühn S., Pieper D. (2019). Single screening versus conventional double screening for study selection in systematic reviews: A methodological systematic review. BMC Med. Res. Methodol..

[73] Mateen F.J., Oh J., Tergas A.I., Bhayani N.H., Kamdar B.B. (2013). Titles versus titles and abstracts for initial screening of articles for systematic reviews. Clin. Epidemiol..

[74] Venancio Rde A., Alencar F.G., Zamperini C. (2009). Botulinum toxin, lidocaine, and dry-needling injections in patients with myofascial pain and headaches. Cranio.

[75] Venâncio Rde A., Alencar F.G., Zamperini C. (2008). Different substances and dry-needling injections in patients with myofascial pain and headaches. Cranio.

[76] Rezaeian T., Mosallanezhad Z., Nourbakhsh M.R., Noroozi M., Sajedi F. (2020). Effects of Dry Needling Technique Into Trigger Points of the Sternocleidomastoid Muscle in Migraine Headache: A Randomized Controlled Trial. Am. J. Phys. Med. Rehabil..

[77] Togha M., Bahrpeyma F., Jafari M., Nasiri A. (2020). A sonographic comparison of the effect of dry needling and ischemic compression on the active trigger point of the sternocleidomastoid muscle associated with cervicogenic headache: A randomized trial. J. Back Musculoskelet. Rehabil..

[78] Hesse J., Møgelvang B., Simonsen H. (1994). Acupuncture versus metoprolol in migraine prophylaxis: A randomized trial of trigger point inactivation. J. Intern. Med..

[79] Karakurum B., Karaalin O., Coskun O., Dora B., Uçler S., Inan L. (2001). The ‘dry-needle technique’: Intramuscular stimulation in tension-type headache. Cephalalgia.

[80] Mousavi-Khatir S.R., Fernández-de-Las-Peñas C., Saadat P., Javanshir K., Zohrevand A. (2022). The Effect of Adding Dry Needling to Physical Therapy in the Treatment of Cervicogenic Headache: A Randomized Controlled Trial. Pain Med..

[81] Gildir S., Tüzün E.H., Eroğlu G., Eker L. (2019). A randomized trial of trigger point dry needling versus sham needling for chronic tension-type headache. Medicine.

[82] Kamali F., Mohamadi M., Fakheri L., Mohammadnejad F. (2019). Dry needling versus friction massage to treat tension type headache: A randomized clinical trial. J. Bodyw. Mov. Ther..

[83] Lonzar G., Abuín-Porras V., Del-Blanco-Muñiz J.A., González-de-la-Flor Á., García-Pérez-de-Sevilla G., Domínguez-Balmaseda D. (2023). Efficacy of invasive techniques in physical therapy for migraine treatment and prevention: A systematic review of randomized controlled trials. Rev. Assoc. Med. Bras..

[84] France S., Bown J., Nowosilskyj M., Mott M., Rand S., Walters J. (2014). Evidence for the use of dry needling and physiotherapy in the management of cervicogenic or tension-type headache: A systematic review. Cephalalgia.

[85] MacPherson H., Altman D.G., Hammerschlag R., Youping L., Taixiang W., White A., Moher D. (2010). Revised STandards for Reporting Interventions in Clinical Trials of Acupuncture (STRICTA): Extending the CONSORT statement. J. Evid. Based Med..

[86] Patra R.C., Mohanty P., Gautam A.P. (2018). Effectiveness of C1-C2 Sustained Natural Apophyseal Glide Combined with Dry Needling on Pressure Point Threshold and Headache Disability in Cervicogenic Headache. Asian J. Pharm. Clin. Res..

[87] Patra R.C., Gautam A.P., Mohanty P. (2017). Effectiveness of dry needling on pain and range of motion in patients with cervicogenic headache. Int. J. Innov. Sci. Res. Technol..

[88] Brady S.R. (2015). Utilizing and Adapting the Delphi Method for Use in Qualitative Research. Int. J. Qual. Methods.

[89] Diamond I.R., Grant R.C., Feldman B.M., Pencharz P.B., Ling S.C., Moore A.M., Wales P.W. (2014). Defining consensus: A systematic review recommends methodologic criteria for reporting of Delphi studies. J. Clin. Epidemiol..

[90] Holey E.A., Feeley J.L., Dixon J., Whittaker V.J. (2007). An exploration of the use of simple statistics to measure consensus and stability in Delphi studies. BMC Med. Res. Methodol..

[91] Legge D. (2014). A History of Dry Needling. J. Musculoskelet. Pain.

[92] Kandeel M., Morsy M.A., Al Khodair K.M., Alhojaily S. (2024). Dry needling techniques as a treatment for improving disability and pain in patients with different types of headache: A systematic review and meta-analysis. Complement. Ther. Med..

[93] Fernández-de-Las-Peñas C., Dommerholt J. (2018). International Consensus on Diagnostic Criteria and Clinical Considerations of Myofascial Trigger Points: A Delphi Study. Pain Med..

[94] Monti-Ballano S., Márquez-Gonzalvo S., Lucha-López M.O., Ferrández-Laliena L., Vicente-Pina L., Sánchez-Rodríguez R., Tricás-Vidal H.J., Tricás-Moreno J.M. (2024). Effects of Dry Needling on Active Myofascial Trigger Points and Pain Intensity in Tension-Type Headache: A Randomized Controlled Study. J. Pers. Med..

[95] Ge H.Y., Arendt-Nielsen L. (2011). Latent myofascial trigger points. Curr. Pain Headache Rep..

[96] Palacios-Ceña M., Ferracini G.N., Florencio L.L., Ruíz M., Guerrero Á.L., Arendt-Nielsen L., Fernández-de-Las-Peñas C. (2017). The Number of Active But Not Latent Trigger Points Associated with Widespread Pressure Pain Hypersensitivity in Women with Episodic Migraines. Pain Med..

[97] Murillo C., Cerezo-Téllez E., Torres-Lacomba M., Pham T.Q., Lluch E., Falla D., Vo T.T. (2024). Unraveling the Mechanisms Behind the Short-Term Effects of Dry Needling: New Insights From a Mediation Analysis With Repeatedly Measured Mediators and Outcomes. Arch. Phys. Med. Rehabil..

[98] Barrett D., Heale R. (2020). What are Delphi studies?. Evid. Based Nurs..

[99] Green R.A. (2014). The Delphi Technique in Educational Research. Sage Open.

